# Mechanisms and treatment of atherosclerosis: focus on macrophages

**DOI:** 10.3389/fimmu.2024.1490387

**Published:** 2024-11-06

**Authors:** LingNa Zhang, JiaWei Li, YuShun Kou, LuFan Shen, Hong Wang, YiYuan Wang, Ruiling Ma, Tao Wu, Xin Yang, YuanHui Gu, Lin Yi

**Affiliations:** ^1^ School of Traditional Chinese and Western Medicine, Gansu University of Chinese Medicine, Lanzhou, Gansu, China; ^2^ Department of General Surgery, Gansu Provincial Hospital, Lanzhou, Gansu, China; ^3^ First School of Clinical Medical, Gansu University of Chinese Medicine, Lanzhou, Gansu, China; ^4^ Chronic Disease Laboratory, Gansu University of Traditional Chinese Medicine, Lanzhou, Gansu, China

**Keywords:** atherosclerosis, polarization of M1/M2 macrophages, targeted macrophage therapy, formation of atherosclerosis, source of macrophages

## Abstract

Macrophages are the basic mediators and coordinators of various types of chronic inflammation and play a crucial role in the formation and development of atherosclerosis (AS). In the complex microenvironment of atherosclerotic plaques, macrophages of different sources are exposed to different signal stimuli and thus polarized into various subpopulations. Various types of macrophages with predominantly M1 and M2 phenotypes also play different regulatory roles in the initiation and progression of AS. Lipid-lowering drugs, mainly statins, are widely used in clinical practice, but the adverse reactions are obvious and there is a lack of personalized treatment. Emerging targeted macrophage and Traditional Chinese medicine (TCM)-related therapies can regulate the cellular microenvironment, inhibit the polarization of M1 macrophages, and promote the activation of M2 macrophages, providing new ideas for the prevention and treatment of AS.

## Highlights

- Macrophage phenotypes and their roles are different, and the different phenotypes can be transformed into each other when the cellular microenvironment is changed, affecting the development of AS.
- The main clinical lipid-lowering drugs for AS: Stains have a large impact of adverse effects that should not be ignored; cholesterol uptake inhibitors are poorly effective when used singly; and PCSK9 inhibitors are not easy to use on a large scale because of their high therapeutic costs.
- Therapies targeting macrophages, including TCM, can modulate macrophage polarization thereby improving inflammation, modulating various lipids (including HDL), and ultimately suppressing AS.

## 1 Introduction

Cardiovascular disease (CVD) is currently the leading cause of death in China, and the significant increase in the incidence of atherosclerotic cardiovascular disease (ASCVD) is one of the characteristics of the prevalence and evolution of CVD in Chin (1). ASCVD includes ischemic heart disease (IHD) and ischemic stroke, of which the mortality rate of IHD is higher than that of ischemic stroke and the increase is faster, and the morbidity and mortality rates of IHD and ischemic stroke vary according to age, gender, and region (2, 3). AS usually leads to a variety of serious acute CVDs such as myocardial infarction, cardiomyopathy, heart failure, and arrhythmias and is the root cause of IHD (4). Therefore, understanding the pathogenesis of AS and improving its therapeutic methods are essential for the diagnosis and treatment of ASCVD.

Macrophages are key effector cells of the immune system, and macrophage polarization refers to the state in which macrophages are activated in a certain time and space. Based on the highly heterogeneous phenotype and function of macrophages, they are generally categorized into M1 and M2 types (5). Macrophages are essential mediators and coordinators of various types of chronic inflammation and are the main participants in a wide range of diseases, such as autoimmune disorders (6), aging (7), cancer (8), and many CVDs (9), and especially play a crucial role in the formation and development of AS (10, 11).

## 2 Source of macrophages

**Figure 1:**
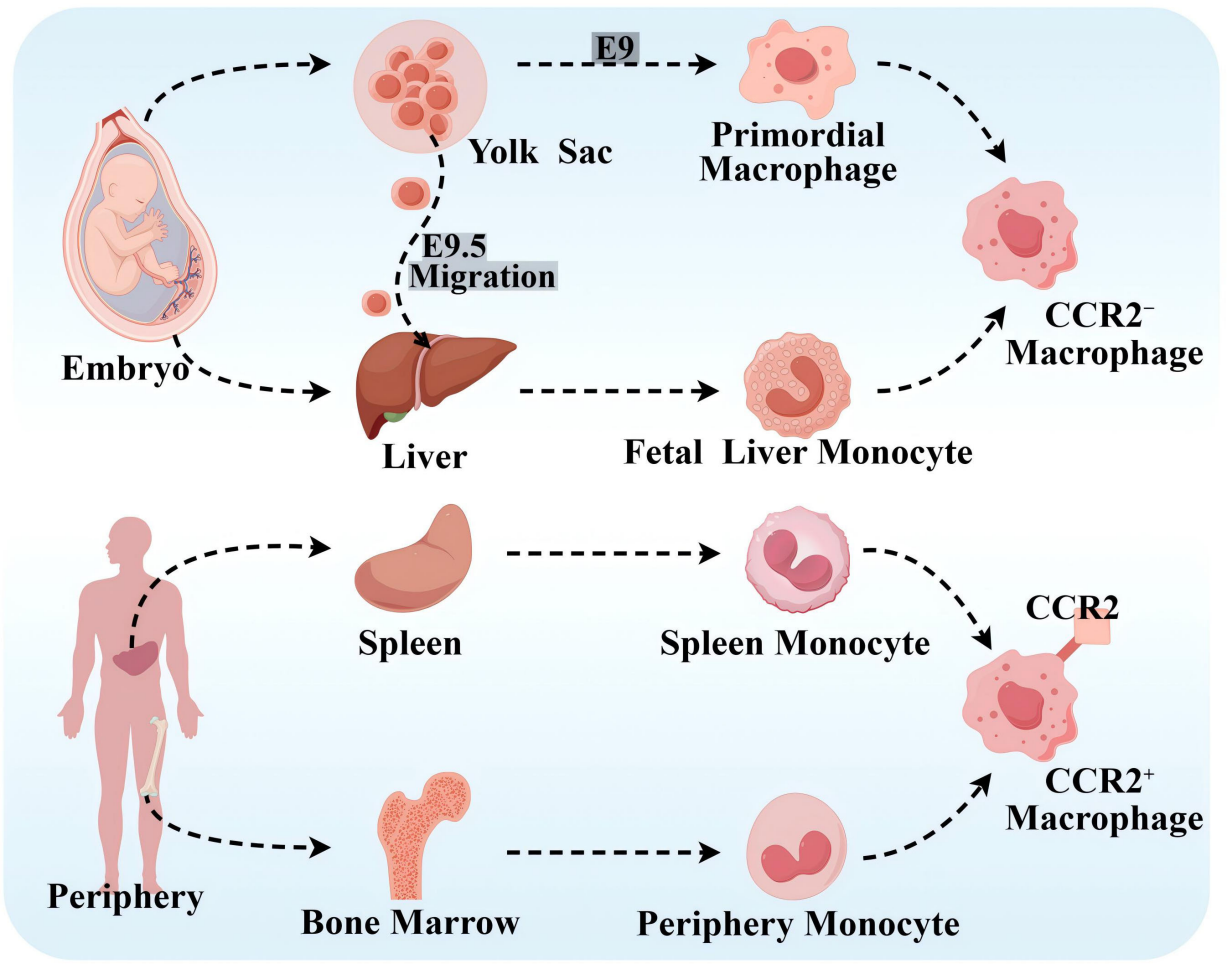
Source of macrophages. CCR2^−^ macrophages originate from the YS and fetal monocyte progenitor cells. Primary erythroid myeloid progenitor cells (EMPs) in the YS product and differentiate into primitive macrophages at embryonic day 9 (E9). Late EMPs migrate to the fetal liver at E9.5, where they develop into fetal liver monocytes, and both of the above cells subsequently migrate into all fetal tissues as both tissue-resident macrophages. CCR2^+^ macrophages originate from peripheral hematopoietic progenitor cells, such as the bone marrow and spleen. By Figdraw.

Based on lineage tracing, flow cytometry, and cell surface expression of CC chemokine receptor 2(CCR2), it has been demonstrated that there are different subpopulations of macrophages in human body containing CCR2^−^ macrophages and CCR2^+^ macrophages (9), which is presented in **Figure 1**. CCR2^−^ macrophages, derived from yolk sac (YS) and fetal monocyte progenitor cells, belong to the group of tissue macrophages that are established during the embryonic period and are independent of blood monocytes. After its settlement in the corresponding target organ, it is maintained by localized proliferation and is not dependent on input supplementation from surrounding monocytes. CCR2^−^ macrophages are long-lived, enriched with genes that orchestrate the potential for tissue repair, and involved in many forms of tissue remodeling such as coronary artery development, vasodilatation, and cardiac tissue repair, and they also show potent pro-angiogenic activity (12, 13). CCR2^+^ macrophages originate from hematopoietic progenitor cells in the periphery, including the bone marrow and spleen, and are maintained under homeostatic and inflammatory conditions by a range of mechanisms, including monocyte recruitment and cell proliferation (14). CCR2^+^ macrophages are closely associated with inflammation and accumulate in areas of scarred or fibrotic tissue, and rare CCR2^+^ monocytes in the cardiac region are also found only in the vicinity of blood vessels located in areas of dense fibrosis (12). Cell proliferation serves as an important mechanism for cell maintenance in each macrophage subpopulation, and CCR2^+^ macrophages show stronger proliferative capacity compared with CCR2^−^ macrophages.

At homeostasis, macrophages are maintained primarily by localized proliferation. Before macrophage depletion, embryonic-established tissue macrophages are independent of peripheral monocytes. However, during macrophage depletion or tissue inflammation, they are maintained mainly by monocyte recruitment, and tissue-resident macrophages also expand numerically through proliferation and even become the dominant macrophage population. Notably, CCR2^−^ macrophages do not represent all tissue-resident macrophages, and CCR2^+^ macrophages did not represent all non-tissue-resident macrophages (15). In addition, it has been shown that the C–C motif chemokine ligand 2 (CCL2)–CCR2 axis effectively promotes monocyte mobilization in the bone marrow and that CCL2 mainly acts on “classical” monocytes expressing CCR2 and recruits circulating monocytes to atherosclerotic plaques (16, 17). In addition, there are no studies that clearly show the correlation between tissue-resident macrophages, or the expression of CCR2 on the surface of macrophages and M1- and M2-type macrophages.

## 3 Polarization of macrophages and AS

AS is a lipid-driven chronic inflammatory disease of the large and medium-sized arterial wall characterized by the formation or even shedding of “plaques” rich in lipids, extracellular matrix, vascular smooth muscle cells (SMCs), and immune cells. Inflammation is one of the important driving factors for the occurrence and development of AS (18) and runs through the whole process of the disease (19). Macrophages are a group of immune cells with high heterogeneity, diversity, and plasticity. In the complex microenvironment of atherosclerotic plaques, macrophages are exposed to different signaling stimuli and thus polarize into different subpopulations with diverse gene and protein expression patterns (20). There are many different macrophage subtypes in atherosclerotic plaques, including M1, M2, and other types of macrophages.

### 3.1 Mechanisms of AS formation

It is generally accepted that high-risk factors, such as hyperlipidemia, smoking, hypertension, obesity, history of heart disease, and diabetes (21), alter the role of the vascular endothelial cells (ECs) semipermeable barrier and affect its ability to regulate the exchange of fluids, nutrients, and metabolites (22). This results in damage to the vascular endothelium, which in turn advances AS. Among them, the formation of thrombosis is caused by certain inflammation, and it will further promote inflammation (23).

Impaired EC function is activated to allow low-density lipoprotein cholesterol (LDL-C) to enter the endangium, where it is modified to oxidized LDL-C (OX-LDL) by free radicals secreted by recruited monocytes. OX-LDL activates the expression of chemokines and adhesion factors (monocyte chemoattractant protein interleukin (MCP)-1, interleukin (IL)-8, vascular cell adhesion molecule (VCAM)-1, endothelial cell (E/P) selection) in ECs and recruits circulating monocytes. OX-LDL recruits more immune cells (T cells, B cells, mast cells, and other immune cells) to the lesion, which together promote plaque formation (24, 25). Recruited monocytes in a local microenvironment enriched with growth factors and pro-inflammatory cytokines subsequently differentiate predominantly into M1 macrophages (26). Macrophages, especially M1 macrophages and SMC-derived macrophages, rapidly recognize and phagocytose OX-LDL via scavenger receptors on their surface, transforming it into foam cells that cause the earliest AS lesions (27, 28). As the disease progresses, accumulation of foam cells, localized necrosis, and fibrous cap formation lead to the formation of stable or unstable plaques. In the late stage of atherosclerosis, plaque instability eventually leads to plaque rupture, hemorrhage, and thrombosis due to hemodynamic changes, stress and inflammatory responses (21).

**Figure 2:**
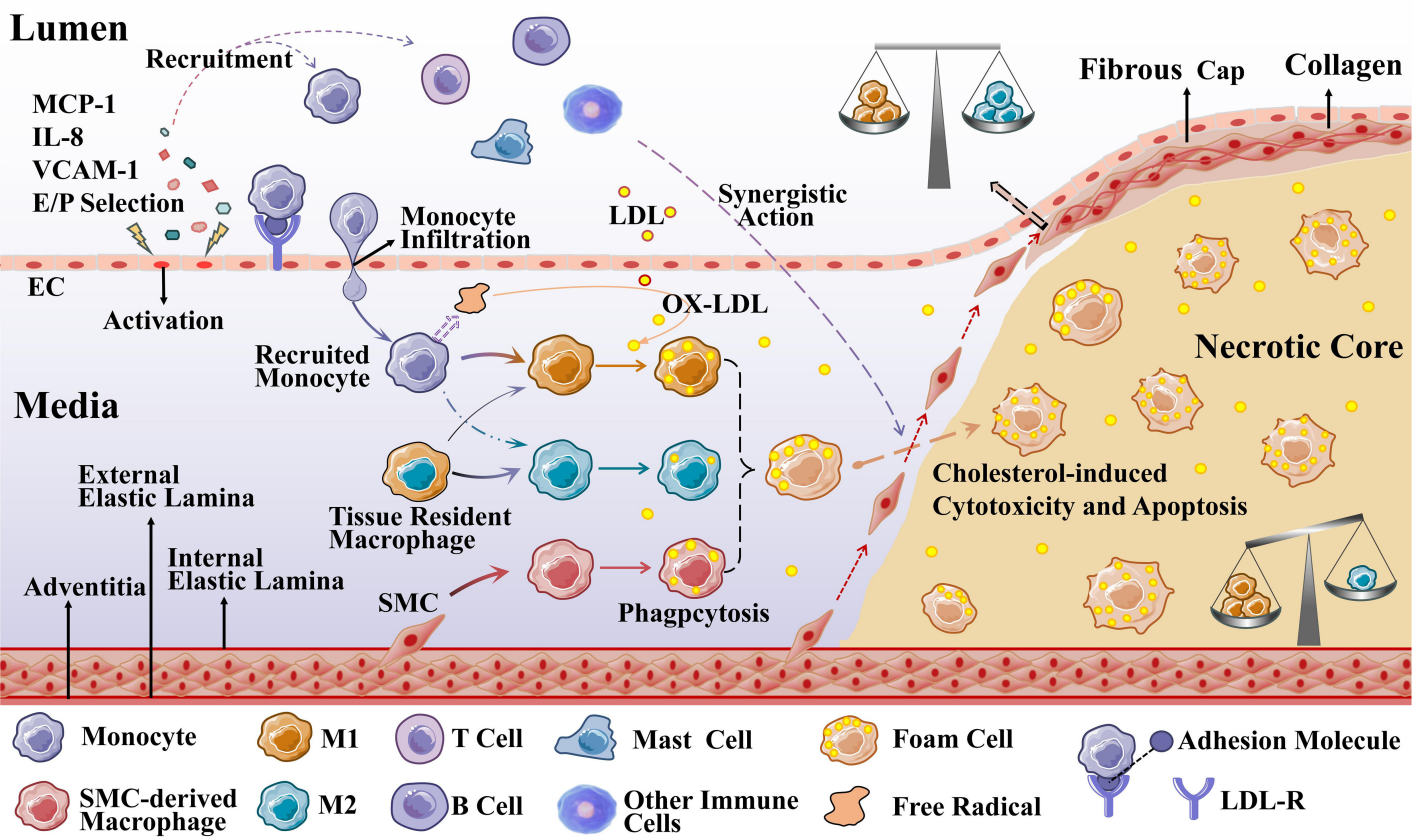
Macrophages during the development of AS. Recruited monocytes (primarily) and tissue-resident macrophages polarize into M1 macrophages (mainly) and M2 macrophages to phagocytose OX-LDL, and SMCs can also differentiate into macrophage-like cells to phagocytose OX-LDL (M2 macrophages are less capable of phagocytosis). When these three types of cells phagocytose an excessive amount of OX-LDL overloaded, they transform into foam cells.

Atherosclerotic plaques are composed of extracellular lipid particles, foam cells, and debris that accumulate within the intima of the arterial wall to form a lipid or necrotic core. The core is encapsulated by a layer rich in collagen fibers, elastin fibers, SMCs, and extracellular matrix called the fibrous cap (29). The ratio of M1 to M2 varies at different stages and locations of AS. Plaques tend to stabilize when M2 macrophages predominate; plaques stability decrease when M1 macrophages infiltrate. Especially the presence of M1 macrophages in the most unstable plaque shoulder will increase necrotic core formation and plaque fragility. In general, there were far more M1 macrophages than M2 in the core; in the region of the fiber cap, the ratio of the two subtypes was similar (30), which is presented in **Figure 2**.

### 3.2 M1 macrophage and AS

Typically activated macrophages, M1 macrophages, are a phenotype characterized by pro-inflammation (31). M1 macrophages focus on the release of inflammatory mediators, killing and clearance of pathogenic microorganisms, and tumor-killing activity. During AS development, macrophages are stimulated by cholesterol crystals, interferon-γ (IFN-γ), lipopolysaccharide (LPS), ox-LDL, Toll-like receptor (TLR) ligands, and the remaining proinflammatory cytokines and are activated to exhibit predominantly pro-inflammatory M1 macrophages (32). Therefore, M1 macrophages secrete pro-inflammatory cytokines such as tumor necrosis factor-α (TNF-α), IL-1β, IL-6, and IL-12, as well as nitric oxide (NO) and reactive oxygen species (ROS) (33). Among them, IFN-γ is the main cytokine associated with M1 activation and a major product of T helper (Th)1 cells (34). LPS promotes the expression and secretion of pro-inflammatory cytokines (e.g., IFN-β, IL-12, TNF, IL-6, and IL-1β), chemokines (e.g., CCL2, CXC chemokine ligand (CXCL) 10, and CXCL11), and antigen-presenting molecules (e.g., major histocompatibility complex (MHC) members, costimulatory molecules, and antigen-processing peptidases) (35). In addition, granulocyte macrophage colony-stimulating factor (GM-CSF) is the newest member of the M1 class of stimulators. GM-CSF is produced by a variety of cells, including macrophages and thin-walled cells, and induces the secretion of the cytokines of IL-6, IL-8, G-CSF, M-CSF, TNF, and IL-1β by monocytes and macrophages, but to a lesser extent than LPS (5).

Functionally, M1 macrophages participate in pathogen clearance and induce tissue damage by activating the nicotinamide adenine dinucleotide phosphate (NADPH) oxidase complex, which generates ROS. Simultaneously, M1 macrophages express chemokine receptor ligands, such as CXCL-9, CXCL-10, and CXCL-5, to promote recruitment of Th1 cells and natural killer cells to generate a sustained inflammatory response that is critical for elimination of cellular pathogens. In the microecological environment of AS, pro-inflammatory M1 macrophages cause persistent inflammation with enhanced phagocytosis and migration, thus sustaining damage to surrounding tissues (33). In addition, enhanced glycolysis and tricarboxylic acid cycle (TCA) cycle blockade in LPS-stimulated macrophages can both promote a maximal inflammatory response and terminate it later. Compared with glycolysis, IL-4-stimulated macrophages are more dependent on fatty acid oxidation (FAO) (36).

### 3.3 M2 macrophage and AS

Alternately activated macrophages, M2 macrophages, are a predominantly pro-inflammatory phenotype, reduce plaque size, and enhance plaque stability (30). M2 macrophages focus on suppression of inflammation, removal of cellular debris and apoptotic cells, and promotion of tissue repair and fibrosis, hepatobiliary regeneration, matrix degradation and repair, and tissue reconstruction (33). It can be polarized by a variety of stimulatory factors, including cytokines (IL-4, IL-10, and IL-13), glucocorticoids, immune complexes (ICs), and LPS (37).

Functionally, activated M2 macrophages produce anti-inflammatory cytokines (e.g., IL-10, IL-4, TNF-β) and chemokines (e.g., CCL17, CCL22, and CCL24), thereby initiating functional anti-inflammatory regulatory mechanisms, which play an important role in suppressing inflammation and repairing damaged tissues. Among them, IL-4 type I and type II receptors activate signal transducer and activator of transcription (STAT) 6, which in turn activates the transcription of genes typical of M2 macrophage polarization (34). M2 macrophages exhibit weak capacity of lipid accumulation due to low expression of liver X receptor α (LXRα), ATP-binding cassette transporter protein 1 (ABCA1), and apolipoprotein E (ApoE). Enhanced cytokinesis in AS lesions induced by increased M2 macrophage polarization shows another role for M2, so that more M2 macrophages are required for plaque regression. M2 macrophages are classified into four subtypes, M2a, M2b, M2c, and M2d, which have different effects on AS (26).

M2a macrophage, induced by IL-4 and IL-13, has potent anti-inflammatory properties, is characterized by poor phagocytosis and inhibition of pro-inflammatory cytokine release, but is insensitive to inflammatory stimuli and is mainly involved in wound healing, tissue remodeling, and angiogenesis. Thus, M2a is less efficient than M1 in inducing antigen presentation, generating toxic oxygen and nitrogen radicals, and killing intracellular pathogens. M2a cells express high levels of mannose receptor (MR), glucocorticoid receptor (SR), and IL-10 receptor (IL-10R). Polarization of M2a results in the release of pro-fibrotic factors such as TGF-β, insulin-like growth factor (IGF), fibronectin, and inflammatory chemokines such as CCL17, CCL18, CCL22, and CCL24, which contribute to extracellular matrix deposition and contribute to tissue repair and thus have been described as “tissue repair” macrophages (38). In addition, IL-6 expression induces M2a macrophage polarization toward M1/M2b; if IL-6 is inhibited, M1/M2b macrophages are induced to polarize toward M2a, thereby controlling atherosclerotic intimal hyperplasia (39).

M2b macrophages, induced by IC, TLR agonists, and IL-1R ligands, are polarized to release high levels of CCL1, IL-6, IL-1β, TNFα, and TNF superfamily member 14 (TNFSF14). In addition, M2b macrophages express and secrete large amounts of the anti-inflammatory cytokine IL-10 and low levels of the pro-inflammatory factor IL-12, with both inhibitory and promotional effects, termed regulatory macrophages (37). M2b macrophages may inhibit AS by suppressing leukocyte infiltration, and transplanted M2b macrophages may also protect the heart from damage associated with ischemia–reperfusion (40).

M2c macrophage, in a microenvironment enriched with glucocorticoids, IL-10, TGFβ, and prostaglandins E2, are induced to polarize to become M2c macrophage by IL-10R and transcription activator 3 (TAT3) (37), which then secrete high levels of IL-10, TGF-β, and PTX3 proteins to achieve the associated inflammatory regression and tissue repair (38). Both M2b and M2c macrophages have regulatory functions in addition to being antigen-presenting cells, which are characterized by the release of the anti-inflammatory cytokine IL-10. However, both M2b and M2c macrophages have the ability to produce large quantities of pro-inflammatory cytokines, and both exhibit high expression of Mer receptor tyrosine kinase and are efficiently phagocytized, so they are also known as regulatory macrophages.

M2d macrophages represent a new subpopulation of M2 macrophage, also known as tumor-associated macrophage, induced by TLR ligands, A2 adenosine receptor agonists, and IL-6 (37). Moreover, M2d macrophages have been shown to participate in angiogenesis through the expression of vascular endothelial growth factor (VEGF) and IL-10, as well as secrete a certain amount of inducible nitric oxide synthase (iNOS), resulting in a pro-inflammatory effect (38). However, unlike the typical M2 macrophage, M2d macrophages do not express Ym1, Fizz1, or cluster of differentiation (CD) 206. Likewise, IL-10 contributes to M2d polarization and VEGF production.

### 3.4 Other types of macrophages and AS

**Figure 3:**
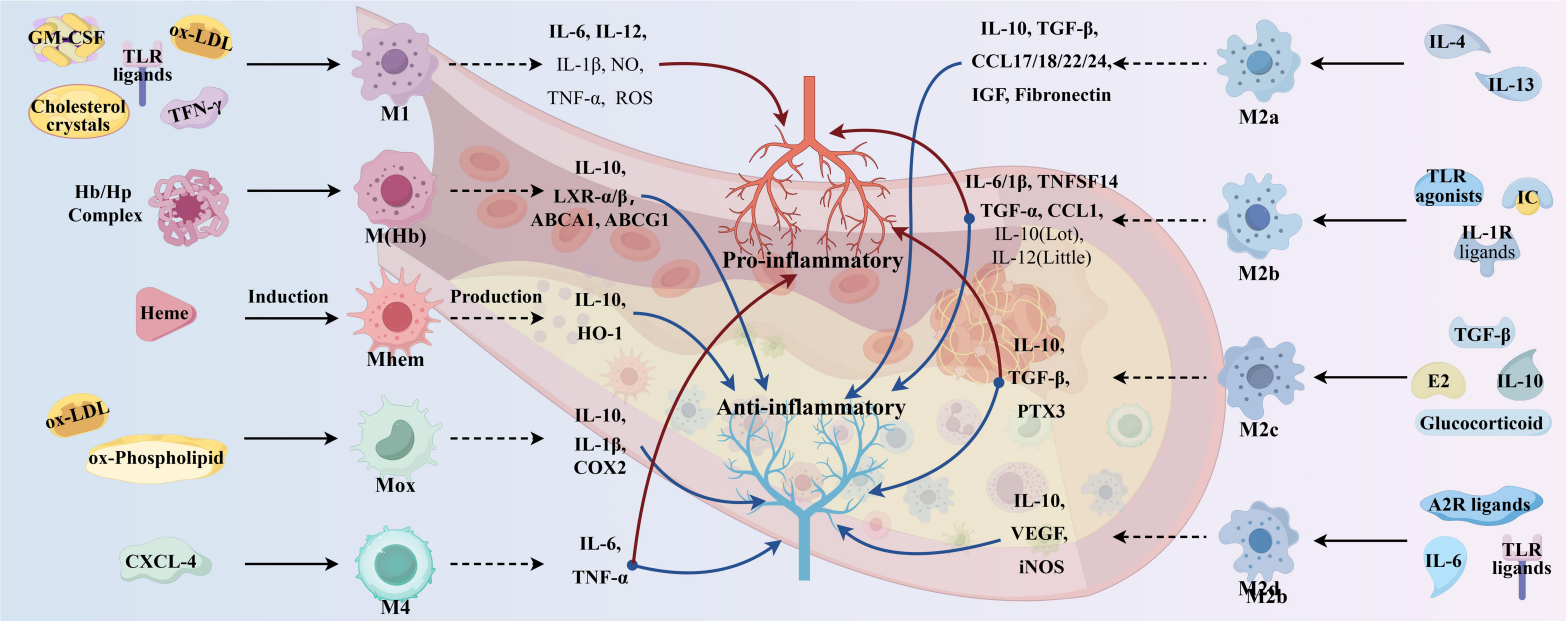
Comparative characteristics of macrophage subtypes. (By Figdraw.).

In addition to macrophages of the M1 and M2 phenotypes, other polarized macrophages were observed in the plaques such as M(Hb), Mhem, Mox, and M4 (32). Comparative characteristics of macrophage subtypes are shown in **Figure 3**. Mox macrophages are induced by oxidized phospholipids and ox-LDL and are characterized by reduced phagocytic activity and chemotaxis, which account for approximately 30% of the total macrophage content of atherosclerotic plaques. In addition, the expression of antioxidant enzymes in Mox macrophages was significantly upregulated by nuclear factor red lineage 2 (NRF 2)-related genes but retained the ability to release the pro-inflammatory cytokines, including IL-10, IL-1β, and cyclooxygenase (COX)-2, thereby exerting an antioxidant effect (30). This suggests that Mox macrophage may also have anti-atherosclerotic and anti-oxidative stress capabilities (41). M4 macrophages are induced to polarization by CXCL4 and express pro-inflammatory cytokines such as IL-6 and TNF-α. However, M4 macrophages express both pro-AS and anti-AS genes and lack the associated expression of CD163, thus having a dual role in AS (30, 32). The phagocytosis of M4 macrophages is inhibited, with a lower expression of scavenger receptors and a higher expression of cholesterol efflux transporter proteins, suggesting a lesser ability to form foam cells (42).

M (Hb) and Mhem macrophages differ from other macrophages in that they coexist at newly formed vessels and the site of hemorrhage in unstable plaques. Intraplaque hemorrhage promotes AS by providing cholesterol-rich erythrocyte membranes and a dual stimulus of oxidized heme and iron. M(Hb) macrophages polarized by hemoglobin (Hb) stimulation typically express high levels of MR and CD163 and are involved in the clearance of the hemoglobin/hemopexin (Hb/Hp) complex after plaque hemorrhage. Following endocytosis of the Hb/Hp complex and erythrocytes, released hemoglobin polarizes macrophages to the Mhem phenotype (42). In intraplaque hemorrhage, M(Hb) and Mhem macrophages recycle erythrocyte remnants and Hb, possibly induced by Hb, albumin, and CD163. M(Hb) macrophages release LXRα, LXRβ, and ABC transporter proteins (ABCA1 and ABCG1) responsible for cholesterol efflux. Mhem macrophages release heme oxygenase-1, which prevents foam cell formation and shows anti-atherosclerotic effects by reducing oxidative damage in plaques. In addition, M(Hb) and Mhem are equally anti-inflammatory, producing anti-inflammatory cytokines such as IL-10, thereby preventing the progression of plaque formation (32).

## 4 Present therapy status of AS

### 4.1 Conventional medical treatment of AS

**Figure 4:**
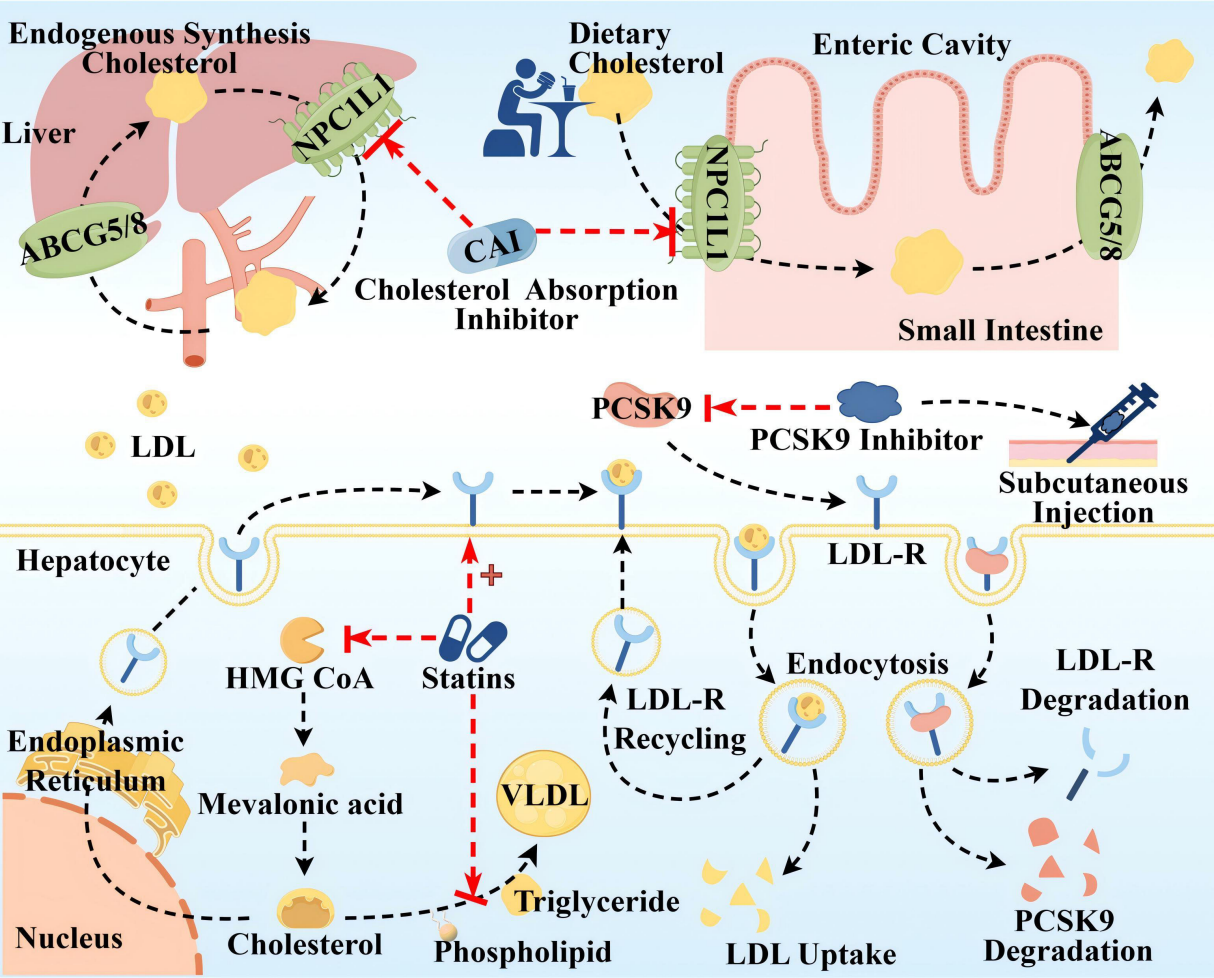
Therapeutic targets and pathway mechanisms of three common lipid-lowering drugs in AS. **The top half** Cholesterol uptake inhibitors mainly inhibit Niemann–Pick C1-like (NPC1L) 1, a highly glycosylated 13-pass membrane receptor protein localized to the apical membrane of intestinal epithelial cells of the brush border of the small intestine and the tubular membranes of hepatocytes, which plays an important role in dietary cholesterol uptake and biliary cholesterol reabsorption. ABCG5/ABCG8 at the same location can translocate unesterified cholesterol back to the intestinal lumen or secrete it directly into the bile. **Lower left** Statins primarily inhibit 3-hydroxy-3-methyl glutaryl coenzyme A (HMG-COA) reductase to reduce the generation of endogenous cholesterol, in addition to upregulating LDL-R synthesis and inhibiting the synthesis of very low-density lipoprotein (VLDL). **Lower right** Left: LDL-C uptake is driven by endocytosis of LDL-R on the surface of hepatocytes. Right: Circulating PCSK9 is captured and presented to the LDLR, which binds to form the PCSK9-LDL-R complex, and after endocytosis, PCSK9 and LDL-R are degraded and reduced. PCSK9 inhibitors injected subcutaneously block LDL-R degradation by inhibiting PCSK9. By Figdraw.

ASCVD is directly associated with elevated levels of LDL-C and ApoB 100, and LDL-C-containing infiltration and retention in the arterial wall are a key initiating event in AS, which triggers an inflammatory response and promotes AS (43). Since cholesterol metabolism is crucial in the pathogenesis of AS, lipid-lowering drugs have long been used as the mainstay of clinical treatment for AS, so this article is only relevant to lipid-lowering drugs. Global clinical guidelines recommend LDL-C as the primary target for lipid control, and statins are recommended as the first-line therapeutic agent for lowering LDL-C levels, in conjunction with cholesterol absorption inhibitor and proprotein convertase subtilisin kexin/type 9 (PCSK9) inhibitors, if necessary (44, 45). The mechanism of the above three classes of drugs is shown in **Figure 4**. Notably, the modulatory effect of statins on inflammation (anti-inflammatory/pro-inflammatory) is somewhat controversial, and statins could induce macrophage polarization into M1-type macrophages, M2-type macrophages, or both M1 and M2, which is related to the cellular microenvironment as well as to the variability of statins (46, 47). It has been shown that monocytes and macrophages are differentially regulated by statins, with monocytes showing no change in response to statins, but macrophages differentiated in the presence of statins remain functionally responsive to inflammatory activation, retaining their monocyte function and maintaining an immune/inflammatory response state (48). In addition, PCSK9 induces pro-inflammatory macrophage activation and does not depend on the mechanism of the LDL receptor (LDL-R) (49, 50), from which it can also be inferred that PCSK9 inhibitors have some anti-inflammatory effects.

These drugs are widely used in clinical practice and have proven efficacy; however, their adverse effects should not be ignored. Adverse effects of statins include abnormalities in liver function, such as elevated transaminases and varying degrees of liver damage. Moreover, statins inhibit mitochondrial function, reduce energy production, and alter muscle protein degradation, resulting in muscle-related symptoms such as muscle pain, muscle weakness, and, in rare cases, rhabdomyolysis (51). In addition, statins have an effect on blood glucose, such as elevated blood glucose, and there is an increased risk of new-onset diabetes with long-term, high-dose statin use (52). Other studies suggest that statins may be the cause of coronary artery calcification and can act as mitochondrial toxins to impair muscle function in the heart and blood vessels (53). Cholesterol absorption inhibitors, such as ezetimibe and hybutimibe, can reduce LDL by approximately 15%–20% when used alone. This is because dietary sources of cholesterol account for 30% of the three cholesterol source pathways, have a single mechanism of action, are less effective than statins, and often need to be used in combination with statins (54). PCSK-9 inhibitors as monoclonal antibodies block PCSK-9 and reduce LDL-C, showing anti-atherosclerotic activity. However, the high cost of treatment limits their larger-scale application. Notably guidelines around the world emphasize the long-term, personalized, and preventive nature of AS drug therapy (55). Therefore, the monotypic and uniform nature of AS medication needs to be somewhat reformed.

### 4.2 Emerging targeted macrophage treatment of AS

#### 4.2.1 Macrophage and inflammatory

As a kind of chronic inflammatory diseases of the arterial wall, the necessity of targeted inflammatory therapy is self-evident, and in recent years, a growing number of studies have shown that AS can be inhibited by modulating M1/M2-type macrophages. One study utilized a mouse model of AS with EC-specific knockout of the transcription factor BACH1 and concluded that deletion of BACH1 attenuates AS by reducing endothelial inflammation (56). Studies have been shown that myeloid-derived CD147 ultimately exacerbates AS by enhancing macrophage infiltration and polarization, leading to enhanced apoptosis and impaired efferocytosis within the plaque, and contributes to plaque instability. It was then concluded that anti-human CD147 antibody prevents AS progression by inhibiting M1-type macrophages polarization, promoting transformation of M2-type macrophages, and thus suppressing inflammation and enhancing efferocytosis (57). It has also been shown that ultraviolet B (UV-B) could inhibit AS by promoting polarization of M2-type macrophages and limiting the inflammatory response in plaques (58).

#### 4.2.2 Macrophage and oxidative stress

Oxidative stress is a type of response in which the degree of oxidation is decompensated after the body is stimulated, leading to excessive accumulation of ROS in the body or in cells. This in turn causes cytotoxicity, which leads to tissue damage, and ROS are also one of the secretions of M1-type macrophages. In AS, increased ROS promote oxidative modification of LDL to OX-LDL, leading to endothelial dysfunction, activation, and promotion of inflammatory factor expression (59). In addition, the large amount of ROS released by damaged endothelial cells can polarize M2-type macrophages to M1-type (60). Studies have shown that L-cystathionine can effectively inhibit oxidative stress in macrophages by suppressing free radical production and enhancing antioxidant capacity to inhibit AS (61). Melatonin can efficiently alleviate PM2.5-induced M1-type macrophage polarization and AS by regulating oxidative stress homeostasis (62).

#### 4.2.3 Macrophage and ferroptosis

Ferroptosis is a kind of cell death induced by lipid peroxidation that occurs in the presence of Fe^2+^ or ester oxygenase. When the ferromodulin–ferritin axis, a key mechanism responsible for regulating iron homeostasis, is abnormal, it causes iron overload, lipid peroxidation, and inflammatory responses in macrophages, thereby promoting AS (63). It was confirmed that differentially expressed genes for ferroptosis in plaques (ALOX5 and NCF2) may promote AS by mediating macrophage ferroptosis (64). Therefore, inhibition of ferroptosis in macrophages is a potential target for the treatment of AS. For example, both micheliolide (64) and melatonin (65) upregulate GPX4/xCT by activating the NRF2 pathway, thereby inhibiting macrophages ferroptosis and ultimately inhibiting AS.

#### 4.2.4 Macrophage and apoptosis and efferocytosis

Macrophage apoptosis and efferocytosis play key roles in the development of AS. Macrophage apoptosis occurs at all stages of AS, and efferocytosis is the process of phagocytosis and removal of apoptotic cells from the lesion. Effective efferocytosis inhibits the formation of necrotic cores, leading to a decrease in pro-inflammatory factors released by apoptotic cells and an increase in anti-inflammatory mediators secreted by phagocytes. In advanced lesions, the long-term accumulation of apoptotic cellular debris, coupled with impaired macrophage efferocytosis, exacerbates inflammation and leads to the formation of plaque necrotic cores (57). It has been shown that NADPH oxidases (NOX) 2 has a key role in AS a major source of ROS in macrophages. NOX2 inhibitors stabilize rupture-prone plaques by promoting intraplaque macrophage efferocytosis via MerTK, which is an important macrophage receptor that binds to apoptotic cells to promote efferocytosis (66). LXR, a member of the metabotropic nuclear receptor superfamily, is a regulatory center of lipid metabolism and inflammation. Among them, LXRα stabilizes vulnerable plaques and prevents plaque rupture by ameliorating macrophage endoplasmic reticulum stress, attenuating apoptosis, and promoting efferocytosis (67).

#### 4.2.5 Macrophage and autophagy-lysosomal pathway

Autophagy is an intracellular degradation system in which macroautophagy transports cytoplasmic material to lysosomes via double-membrane autophagosomes, and degradation of other forms of autophagy, including chaperone-mediated autophagy and microautophagy, occurs directly on lysosomes (68). The autophagy-lysosomal pathway in macrophages shows an important protective role in the development of AS by promoting lipid solubilization and efflux (69). Reduced ox-LDL or adiponectin allows lysosomal dysfunction, whereas plaque formation leads to autophagy dysfunction, and impaired autophagy-lysosomal pathways lead to cholesterol crystal accumulation and mitochondrial dysfunction, which ultimately promotes inflammasome hyperactivation, M1-like polarization, and apoptosis thereby promoting AS (70). In addition, activation of the mTOR pathway inhibits autophagy and leads to lysosomal dysfunction, and cholesterol transport from lysosomes to the endoplasmic reticulum is also blocked by the activated mTOR pathway. Targeting the autophagy-lysosomal pathway in macrophages and inhibiting the mTOR pathway could increase M2-type polarization, which stabilizes plaques and blocks M1-type polarization (69–71). Research has shown that arsenic trioxide promotes ROS induction, leading to nuclear translocation of the transcription factor EB and inhibition of the PI3K/AKT/mTOR pathway, which ultimately promotes autophagy of macrophages and inhibits early-stage lesions in AS (71). Overexpression of the autophagy-specific regulator autophagy-related (ATG) 14 in macrophages enhances autophagosome-lysosome fusion, promotes lipid degradation to reduce Ox-LDL-induced apoptosis and inflammation, and ultimately alleviates AS (72). Expression of the macrophage-specific autophagy-related gene, the small heat shock protein (HSPB) 8, is associated with macrophage polarization and inflammatory factors in AS, and down-regulation of HSPB8 may be involved in M2-type macrophage polarization in AS, which may indirectly accelerate AS progression (73).

#### 4.2.6 Macrophage and others

SMC plays an important role in AS through phenotypic switching and is a major source of foam cells. In contrast to macrophages, low levels of lysosomal acid lipase in SMC lead to defects in their lysosomal cholesterol processing (74), thereby increasing cholesterol efflux and promoting AS. Thus, SMC can be a new target for the prevention and treatment of AS (75). It has been shown that all-trans retinoic acid may have a positive therapeutic effect on AS by blocking SMC phenotypic transformation and promoting fibrous cap stability (76).

In addition, dyslipidemia is not only an elevation of LDL-C but also an elevation of triglyceride-rich lipoproteins (TGRL) and a decrease in high-density lipoproteins (HDL). While LDL-lowering therapies may contribute to the overall decline in LDL and obesity, subsequent insulin resistance and a high-carbohydrate diet lead to an increased prevalence of a number of disorders, such as “metabolic syndrome,” which is characterized in part by elevated TGRL (19). Clinical studies have found that HDL levels are negatively associated with the risk of developing ASCVD (77). HDL is primarily responsible for transporting cholesterol from peripheral tissues, including atherosclerotic plaques, to the liver, where it is excreted as bile acids. Activating transcription factor (ATF) 3 in hepatocytes can ultimately inhibit the development of AS by enhancing HDL uptake, inhibiting intestinal cholesterol and lipid absorption, regulating bile acid metabolism, and promoting reverse cholesterol transport in macrophages (78). It has been shown that modification of HDL by reactive dicarbonyl species significantly impairs the anti-AS function of HDL and that dicarbonyl scavenger enhances HDL function, reduces systemic inflammation, increases plaque stability, and slows the progression of AS (79). Therefore, treatments targeting HDL against AS also have great potential.

**Figure 5:**
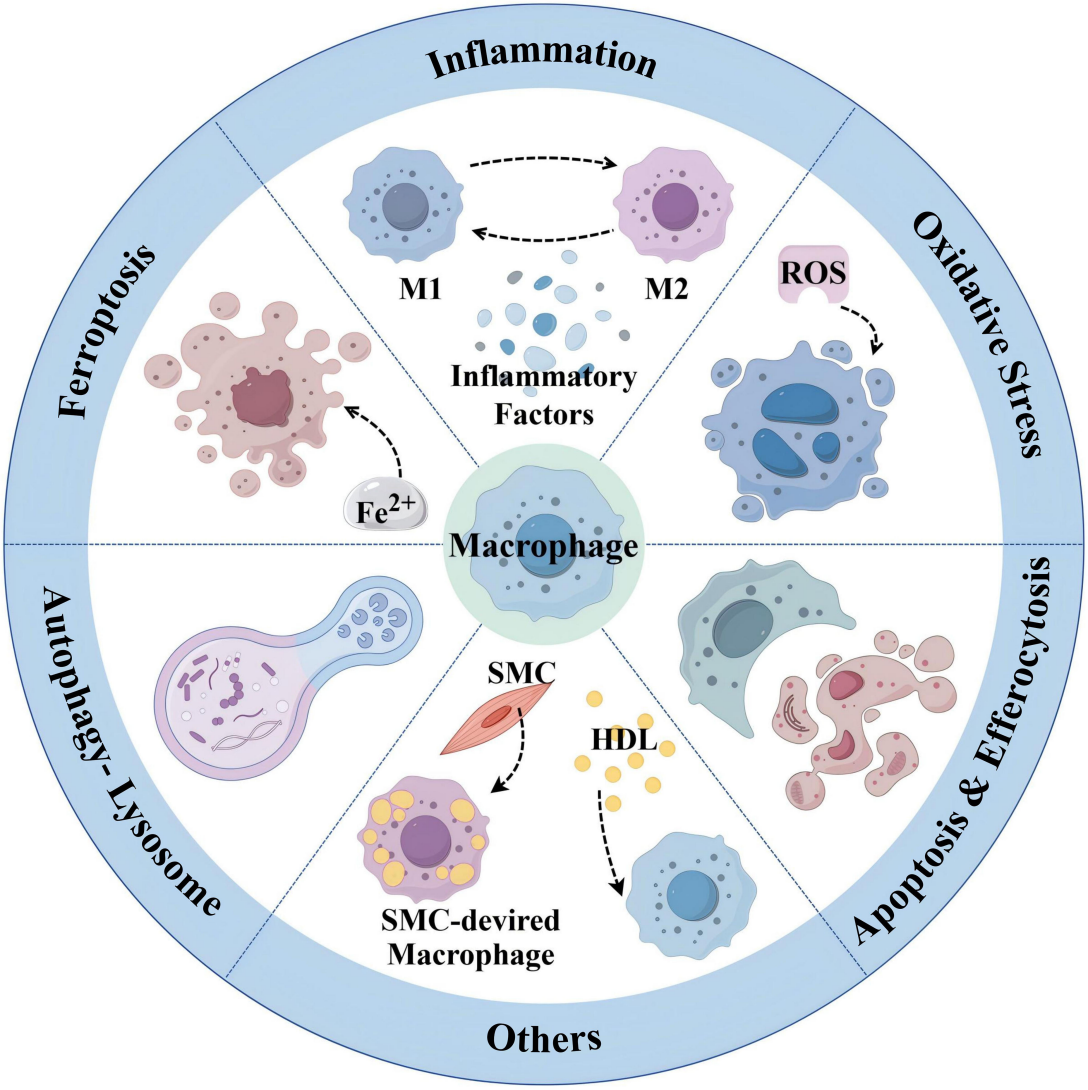
Summary of emerging targeted macrophage treatment of AS (By Figdraw.).

Taken together, all of the above relevant targets and mechanisms provide new reference information for targeting macrophages to prevent and treat AS (see **Figure 5**).

### 4.3 Macrophages and traditional Chinese medicine treatment in AS

**Figure 6:**
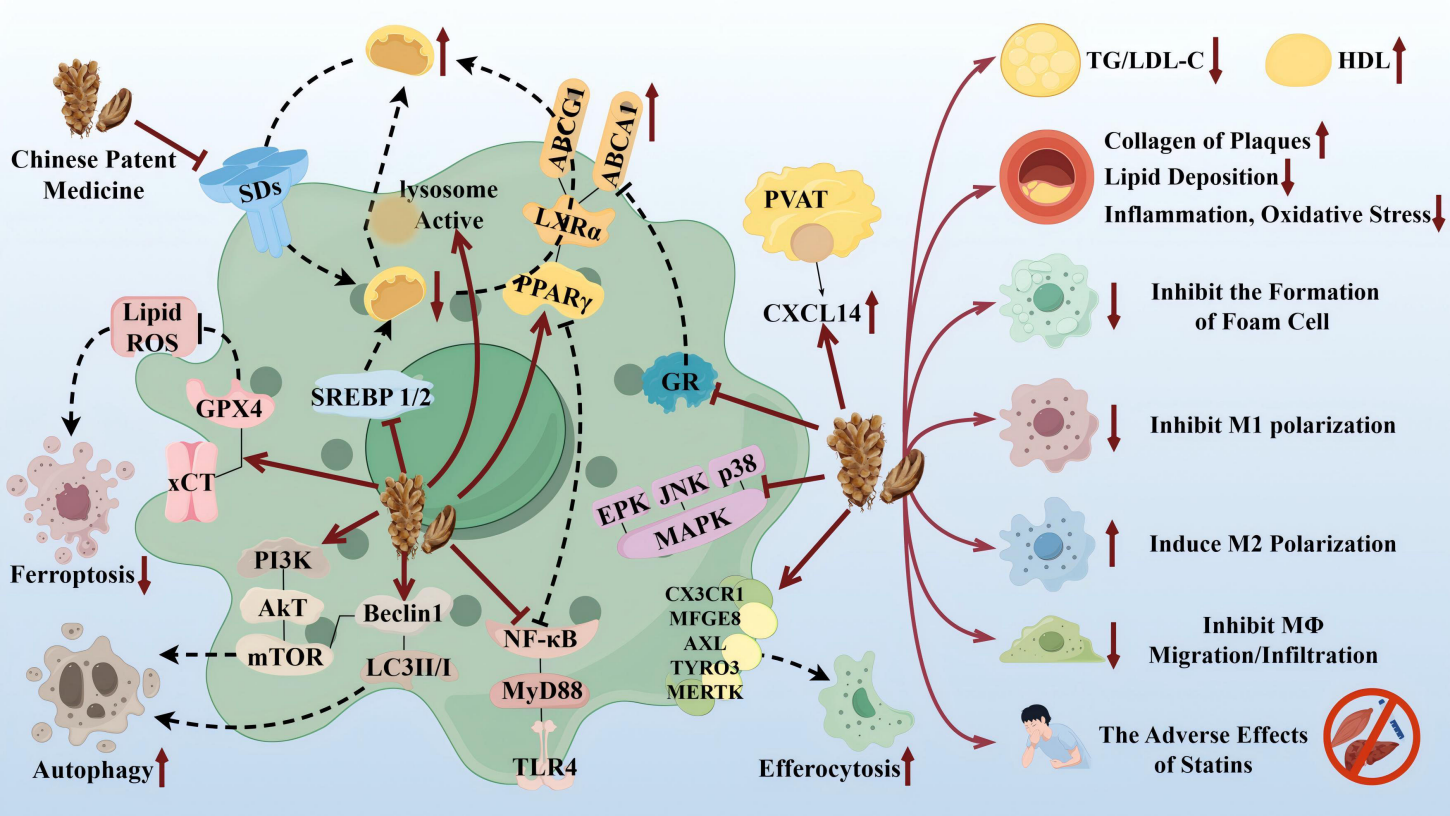
Therapeutic targets and pathway mechanisms of partial TCM for AS. Left: TCM acts on macrophage-related targets and pathways. Right: Summary of commonalities related to TCM treatment. By Figdraw.

TCM has the advantages of multicomponents and multitargets. In recent years, a number of studies have provided new ideas for the prevention and treatment of AS. In this paper, the mechanism of TCM in treating AS through the target of macrophages was explored in three TCM categories: monomer, extract, and compound. The search tool used was *PubMed*, and *AS, Macrophages & TCM* was searched in the *title/abstract*. The details are shown in **Table 1** (herbal monomers/extracts for AS via macrophages) and **Table 2** (herbal compounds for AS via macrophages). Moreover, according to the table, the mechanisms and effects of TCM medicines in treating AS were summarized, and the details are shown in **Figure 6**.

**Table 1:** Herbal monomers/extracts for AS via macrophages.

Herb monomer/extract	Subject	Target/pathway	Effect	Compounds	Ref.
Geniposide (compound of Gardenia jasminoides Ellis )	ApoE^−/−^ mice, 3T3-L1 adipocytes, RAW264.7 macrophages,	(+) CXCL14	Induce M2 polarization of plaque macrophages		(80)
Tanshinone IIA (the major lipid-soluble active ingredient of the Salvia miltiorrhiza)	LDLR^−/−^ mice, RAW264.7 cells	(+) CX3CR1, MFGE8, AXL, TYRO3, MERTK	Regulate efferocytosis of macrophages		(88)
Ganoderma lucidum spore powder (GLSP) and GLSP-derived triterpenes	LDLR^−/−^ mice, RAW264.7, and HASMC cells	(+) ABCA1/G1 (−)RUNX2	Improve cholesterol efflux in MΦ, reducing aortic calcification, inhibited the inflammation, endothelium injury and oxidative stress	Ganoderic acid A, ganoderic acid B, ganoderic acid C6, ganoderic acid G, and ganodermanontriol	(89)
Danhong injection (extracted from Radix Salviae miltiorrhizae and Flos Carthami tinctorii)	Male ApoE^−/−^ mice, RAW264.7 murine MΦ cells	(+) PI3K/AKT insulin pathway	Prevent MΦ lipid accumulation	Tanshinone, tanshinol acid, and safflor yellow	(90)
Ophiopogonis Radix	Male ICR mice, mouse peritoneal MΦ cells	(+) ABCA1, (−) CD36, Lox-1, SREBP2,	Reduce the formation of MΦ foam cells (by uptake, synthesis and increasing efflux), increase antioxidant capacity in MΦ cells	Methylophiopogonanone A and methylophiopogonanone B	(91)
Leech Whitmania pigra	RAW264.7 macrophages	(−) JNK and p38 MAPK pathways, MEKK4, ASK2	Inhibit MΦ migration	The leech hydrolysate and the leech peptide (HE4-1)	(92)
Diterpenoids (one of the major structures of the chemical constituents in plants of the genus Callicarpa)	RAW264.7 cells	(+) PPARγ-LXRα-ABCA1 pathway	Inhibit ox-LDL-induced MΦ foam cell formation by promoting cholesterol efflux	14α-hydroxyisopimaric acid and isopimaric acid (isolated from the flowers of Callicarpa rubella Lindl)	(93)
Ganoderma lucidum spore ethanol extract	Male Japanese white rabbits	(+) LXRα	Promote lipid metabolism(HDL-C↑, TG and LDL-C↓), inhibit foam cell formation, exclude the typical adverse effects of statins	Polysaccharides and triterpenic acid (ganoderic acid A/G/B/C2/I)	(94)
Leech peptide HE-D (synthetic leech peptide)	Mouse RAW264.7 macrophages	(−) NF-κB signaling pathway	Inhibit MΦ migration activity, promote the transformation of macrophages from M1 to M2 subtypes		(81)
Total flavonoids of Engelhardia roxburghiana wall	Male Apoe^−/−^ mice, human monocyte line THP-1 cells	(−) AKT/mTOR	Activate MΦ autophagy, reduce MΦ infiltrations, lipid accumulation, foam cells formation and inflammatory responses	Quercitrin, astilbin, engeletin, quercetin, kaempferol	(95)
Guang Chen Pi (the pericarp of Citrus reticulata Blanco’s cultivars ‘Chachi’)	RAW264.7 macrophages	(+) PPARγ-LXRα-ABCG1/SRB1 pathway (−) SRA1 and CD36, p38 MAPK, ERK1/2, JNK1/2, NF-κB p65 and IKKα/β	Inhibit foam cell formation and lipid accumulation, promote cholesterol efflux, suppress the inflammatory response.	Flavonoids, phenolic acids, limonoids, and coumarins (such as tangeretin, nobiletin, 3-methoxynobiletin, 5¸7¸3′¸4′-tetramethoxyflavone, hesperidin)	(96)

**Table 2:** Herbal compounds for AS via macrophages.

Formula	Subject	Target/pathway	Effect	Compounds	Ref.
Yin-xing-tong-mai tooction	ApoE^−/−^ mice, RAW264.7 murine MΦ cell line	(+) PPARγ-LXRα-ABCA1/ABCG1 pathway	Increase cholesterol efflux of foam cell, and inflammatory response reduction	Genistein, chlorogenic acid, and (-)-catechin	(97)
Qing-Xin-Jie-Yu Granule	ApoE^−/−^ mice, J744A.1 cells	(+) GPX4/xCT signaling pathway	Inhibit MΦ ferroptosis	Salvianolic acid B, ferulic acid, astragaloside IV, berberine, epberberine, coptisine, and palmatine	(98, 99)
Qing-Xue-Xiao-Zhi formula	ApoE^−/−^ mice, RAW264.7 murine MΦ cell line	(−) TLR4/MyD88/NF-κB pathway, (+) PPARγ/LXRα/ABCA1/ABCG1	Facilitate reverse cholesterol transport in MΦ and inhibit MΦ-mediated inflammation	Emodin, curcumin, atractylenolide, polydatin, rhein, and kaempferide	(100)
Jisil Haebaek Gyeji-Tang(Zhi-Shi-Xie-Bai-Gui-Zhi-Tang)	RAW264.7 cells, mouse bone marrow-derived macrophages, zebrafish	(−) MAPK, NF-κB translocation, NO	Reduce inflammatory M1 MΦ polarization	A. macrostemon	(82)
San-wei-tan-xiang capsule	ApoE^−/−^ mice, RAW264.7 cells	(+) Tim4, CD36, lysosomal enzymes	Elevate lysosomal activity in adipose tissue macrophages, enhance MΦ cholesterol efflux	Quinic acid, esculetin, morin and curcumol……	(101)
Shen-Yuan-Dan Capsule	ApoE^−/−^ mice, RAW264.7 cells	(+) Beclin1/LC3II/I (−) PI3K/Akt/mTORC1 signaling pathway	Enhance autophagy, attenuate foam cell formation	Tetrahydropalmatine, harpagoside, salvianolic acid A, salvianolic acid B, and tanshinone IIA.	(102)
Tanyu Tongzhi Formula	Male ApoE^−/−^ mice, primary peritoneal macrophages	(+) PPARγ (−) AKT/ERK signal pathway	Promote alternative (M2) MΦ activation	Hypoxanthine, uracil, adenosine, quercetin, lithospermic acid B, lithospermic acid, tanshinone I	(83)
Xiaoyaosan	Male SD rat, rat peritoneal macrophages (PMs)	(+) ABCA1 (−) Glucocorticoid receptor (GR)	Increase viability and decrease lipid accumulation in corticosterone-induced stress rat PMs		(103)
Xuefu Zhuyu Decoction	ApoE^−/−^ mice, RAW264.7 cell	(−) CX3CL1 and CX3CR1 (+) GDF5	Reduce lipid deposition and MΦ infiltration, increase collagen of plaques	Naringin, isoliquiritin, paeoniflorin, protocatechuic acid, neohesperidin, and ferulic acid	(104, 105)
Danlou Recipe	P407-induced hyperlipidemia mouse, male murine MΦ RAW264.7 cells and murine peritoneal macrophages	(+) LXR pathway	Enrich cytokine–cytokine receptor interaction and chemokine signaling pathways, inhibit the formation of foam cells, promote cholesterol efflux	Gallic acid, puerarin, daidzin, paeoniflorin, ferulic acid, calycosin-7-O-glucoside, naringin, salvianolic acid B, cryptotanshinone, and tanshinone IIA	(106)

Notably, TCM can inhibit the polarization of M1 macrophages and promote the activation of M2 macrophages as a means of delaying the development of AS and enhancing plaque stability. For example, in the table, the herbal monomer geniposide induced M2 polarization in plaques via upregulation of CXCL14 (80); the herbal extract Leech Peptide HE-D inhibited macrophage migratory activity and promoted macrophage transformation from M1 to M2 via the NF-κB signaling pathway (81); the herbal compound Jisil Haebaek Gyeji-Tang inhibits polarization of inflammatory M1-type macrophages by inhibiting MAPK and NF-κB signaling pathways (82); and Tanyu Tongzhi Formula promoted the activation of M2 macrophages by activating the PPARγ signaling pathway and inhibiting the AKT/ERK signaling pathway (83). Thus, macrophage polarization is closely related to cytokine–cytokine receptors, chemokine-related signaling pathways, and inflammation-related MAPK and NF-κB signaling pathways, reflecting the importance of the cellular microenvironment for macrophage polarization and function.

It has been shown that Ganoderma Lucidum Spore Ethanol Extract, via the LXRα-related pathway, can regulate lipid metabolism, in which TG and LDL-C are reduced and HDL-C is elevated, thereby inhibiting foam cell formation and also ruling out the typical adverse effects of statins, such as muscle-related symptoms and liver function abnormalities. Therefore, the targets of TCM are not limited to the reduction of LDL but also target the elevation of HDL, which serves to comprehensively regulate lipids.

## 5 Conclusion and perspective

With further development of the research, three hypotheses of AS generation have been proposed: Response-to-injury hypothesis, Response-to-retention hypothesis, and Oxidative modification hypothesis. Although the key factors for AS initiation differ among the three hypotheses, all emphasize inflammation (especially macrophages) as a necessary and sufficient condition to demonstrate AS development (25). Macrophages play a central role in the development of AS. As one of the major immune cells in the body, they are actively involved in the physiological and pathological processes of *in vivo* immunity, regulating local inflammatory responses and promoting plaque formation and thrombosis through antigen presentation, polarization, and phagocytosis. Therefore, a deeper understanding of the functions of different macrophage phenotypes and the mechanisms behind these functions could help to identify the potential role played by macrophages in the pathogenesis of AS.

In addition, many factors have been associated with the occurrence, progression, and outcome of AS, such as metabolic reprogramming, miRNAs, and epigenetic modifications, all of which influence macrophage polarization (30, 57). Transcription factors, epigenetic landscapes, and microRNA networks are regulated through the expression of multiple layers of related genes, resulting in the dynamic regulation of a complex network of genes that control macrophage polarization and all types of macrophages. These factors strongly influence macrophage polarization, as well as macrophage-mediated inflammatory responses. For example, microRNAs are able to modulate the duration and magnitude of the innate immune responses, participating as part of a feedback loop regulatory mechanism that significantly shapes the inflammatory response and modulates sensitivity to endotoxin to prevent hyperinflammation in macrophages (10).

Notably, smart bio-nanoparticles and drug-controlled release strategies have been a hotspot of research in recent years, and nanotechnology has a promising future in the diagnosis and treatment of AS (84). Compared with traditional drugs, functionalized modification of nanoparticle surfaces using targeted ligand molecules enables nanomedicines to precisely target AS vascular ECs, which can effectively reduce off-target effects (85). Macrophages, which are present in a variety of tissues under homeostatic conditions and polarize into different phenotypes in response to key factors in the cellular microenvironment, are excellent tools for manipulating and targeting therapeutic agents. Among other things, emphasis will be placed on manipulating macrophage signaling pathways and other mechanisms of AS (86). However, effective delivery of therapeutic agents to targeted lesions and cells remains a daunting task. More mature nanotechnologies have been developed and focused on the diagnosis and treatment of atherosclerosis, such as micro-nanobubbles (MNBs). When microbubbles are introduced into the blood stream, the size of the bubbles remains stable, and the smaller the diameter the better the distribution and penetration efficiency, especially for micro- and nano-bubbles. MNBs become extremely promising therapeutic agents by reaching the target site by aggregation or penetration with sufficiently small size and strong properties (87).

In summary, further studies on the specific mechanisms of macrophage polarization in AS will provide a theoretical basis for the development of new therapeutic strategies targeting macrophage polarization. Modulation of macrophage polarization status and promotion of M2-type macrophage function may help to reduce inflammation and improve plaque stability. Also, monitoring of macrophage polarization may be one of the indicators for assessing disease risk and treatment efficacy.

## Glossary

AS: Atherosclerosis
TCM: Traditional Chinese medicine
CVD: Cardiovascular disease
ASCVD: Atherosclerotic cardiovascular disease
IHD: Ischemic heart disease
CCR2: CC chemokine receptor 2
CCL2: C–C motif chemokine ligand 2
YS: Yolk sac
EMP: Erythroid myeloid progenitor cell
E9/9.5: Day 9/9.5
SMC: Smooth muscle cell
EC: Endothelial cell
LDL-C: Low-density lipoprotein cholesterol
OX-LDL: Oxidize LDL
MCP-1: Monocyte chemoattractant protein-1
IL: Interleukin
VCAM-1: Vascular cell adhesion molecule-1
E/P Selection: Endothelial cell Selectin
IFN-γ/β: Interferon-γ/β
LPS: Lipopolysaccharide
TLR: Toll-like receptor
TNF-α: Tumor necrosis factor-α
NO: Nitric oxide
ROS: Reactive oxygen species
Th1 cell: T helper 1 cell
CXCL: CXC chemokine ligand
MHC: Major histocompatibility complex
G-CSF: Granulocyte colony-stimulating factor
M-CSF: Macrophage colony-stimulating factor
NADPH: Nicotinamide adenine dinucleotide phosphate
TCA: Tricarboxylic acid cycle
FAO: Fatty acid oxidation
IC: Immune complexes
STAT6: Signal transducer and activator of transcription 6
LXRα/β: Liver X receptor α/β
ABCA1: ATP-binding cassette transporter protein 1
ApoE/B: Apolipoprotein E/B
MR: Mannose receptor
SR: Glucocorticoid receptor
IL-xR: IL-x receptor
TGF-β: Transforming growth factor-β
IGF: Insulin-like growth factor
TNFSF14: TNF superfamily member 14
TAT3: Transcription activator 3
VEGF: Vascular endothelial growth factor
iNOS: Inducible nitric oxide synthase
CD206/147: Cluster of differentiation 206/147
NRF2: Nuclear factor red lineage 2
COX-2: Cyclooxygenase-2
Hb: Hemoglobin
Hb/Hp Complex: Hemoglobin/hemopexin complex
ABCG1/5/8: ATP-binding cassette (ABC) transporters G1/5/8
PCSK9: Proprotein convertase subtilisin kexin/type 9
LDL-R: Low-density lipoprotein receptor
NPC1L1: Niemann–Pick C1-like 1
HMG-COA: 3-Hydroxy-3-methyl glutaryl coenzyme A
VLDL: Very low-density lipoprotein
UVB: Ultraviolet B
NOX2: NADPH oxidases 2
ATG14: Autophagy-related 14
HSPB8: The small heat shock protein B8
ATF3: Activating transcription factor 3
TGRL: Triglyceride-rich lipoproteins
HDL: High-density lipoproteins.
